# Supplementary material for: Ecological change and conflict reduction led to a social circulatory system in ants
Source: Commun Biol. 2025 Feb 16;8:246. doi: 10.1038/s42003-025-07688-7 (PMC11830068; doi:10.1038/s42003-025-07688-7)
Supplement: Supplementary file 1 — SUPPLEMENTAL MATERIAL [file 42003_2025_7688_MOESM1_ESM.pdf]

Supplementary Information for

Ecological change and conflict reduction led to a social circulatory system in ants

Marie-Pierre Meurville[1], Daniele Silvestro[2,3]*, Adria C. LeBoeuf[1,4]*•

[1] Department of Biology, University of Fribourg, Fribourg, Switzerland

[2] Department of Biological and Environmental Sciences, Gothenburg Global Biodiversity Centre, University of Gothenburg, Gothenburg, Sweden

[3] Department of Biosystems Science and Engineering, ETH Zurich, Klingelbergstrasse 48, 4056 Basel, Switzerland

[4] Department of Zoology, University of Cambridge, Cambridge, United Kingdom

* Authors contributed equally

• Author correspondence: ACL: acl79@cam.ac.uk

Contains:

Figures S1-S8

Tables S1-S5

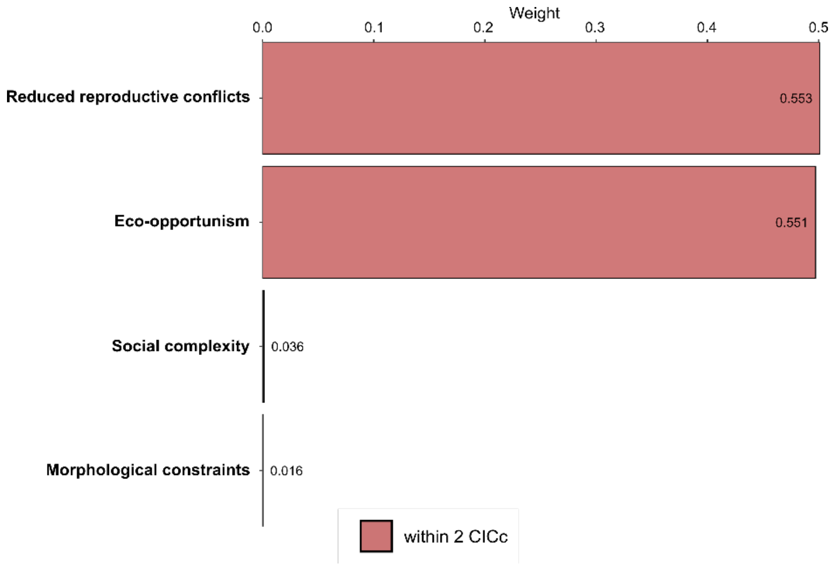

Figure S1 Model statistics. Models are ordered by weight, models in red are indistinguishably good (non-significant p-values, ΔCICc < 2), and were averaged into one summary model.

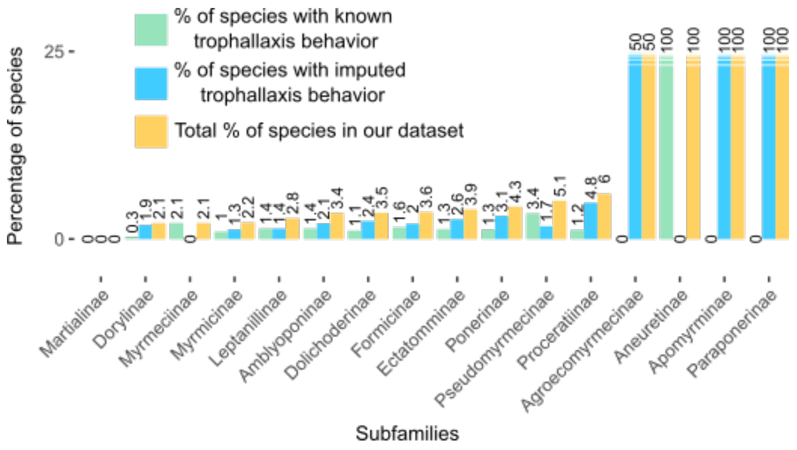

Figure S2 Percentages of species with trophallaxis behavior known (present or absent) from literature (165 species), imputed (252 species), and both (417 species), respective to subfamily diversity.

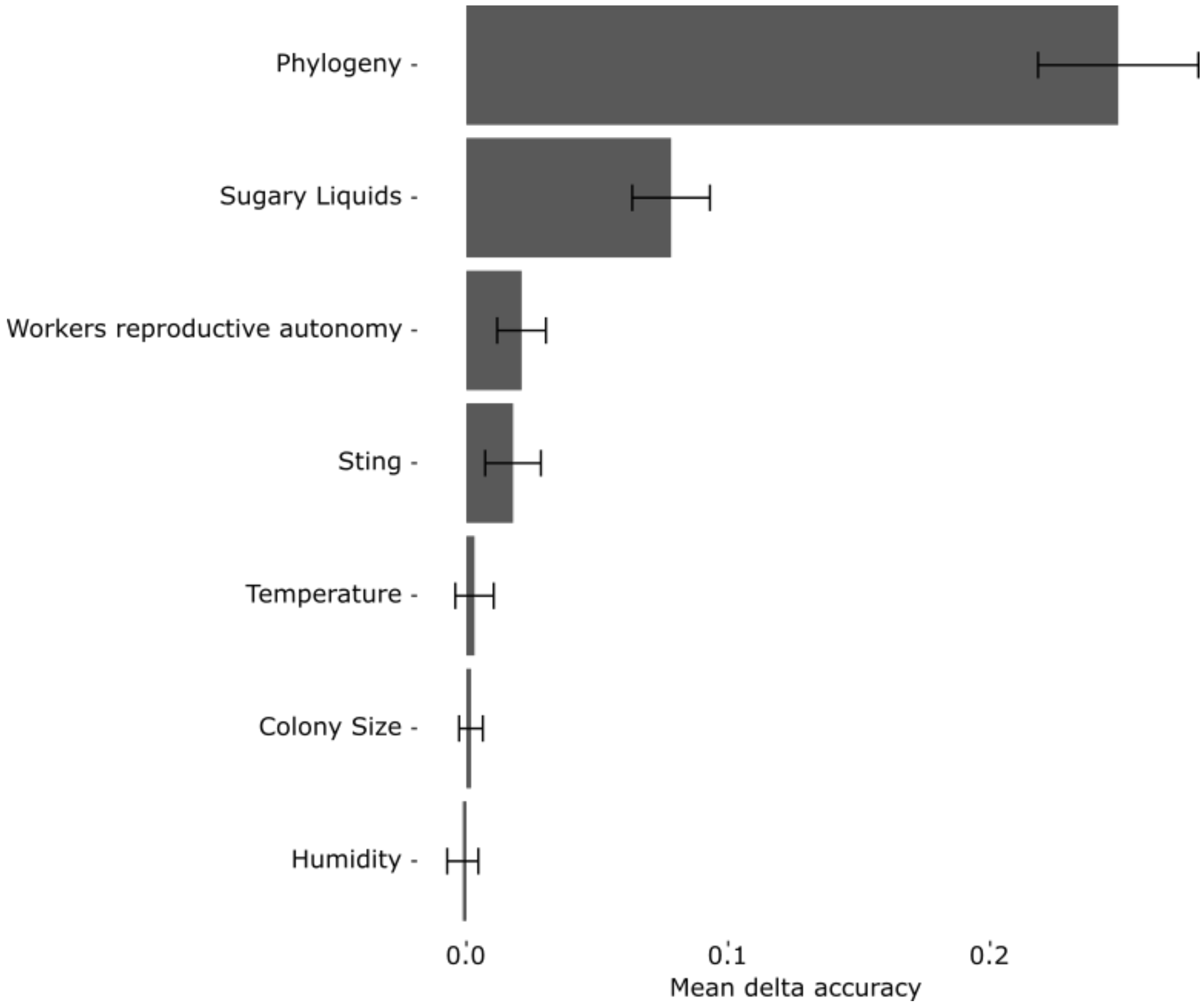

Figure S3 Feature importance from the Bayesian Neural Network model. The bigger the mean delta accuracy, the less accurate the model is when a given feature, or block of features is randomized, the most important for the model the feature is.

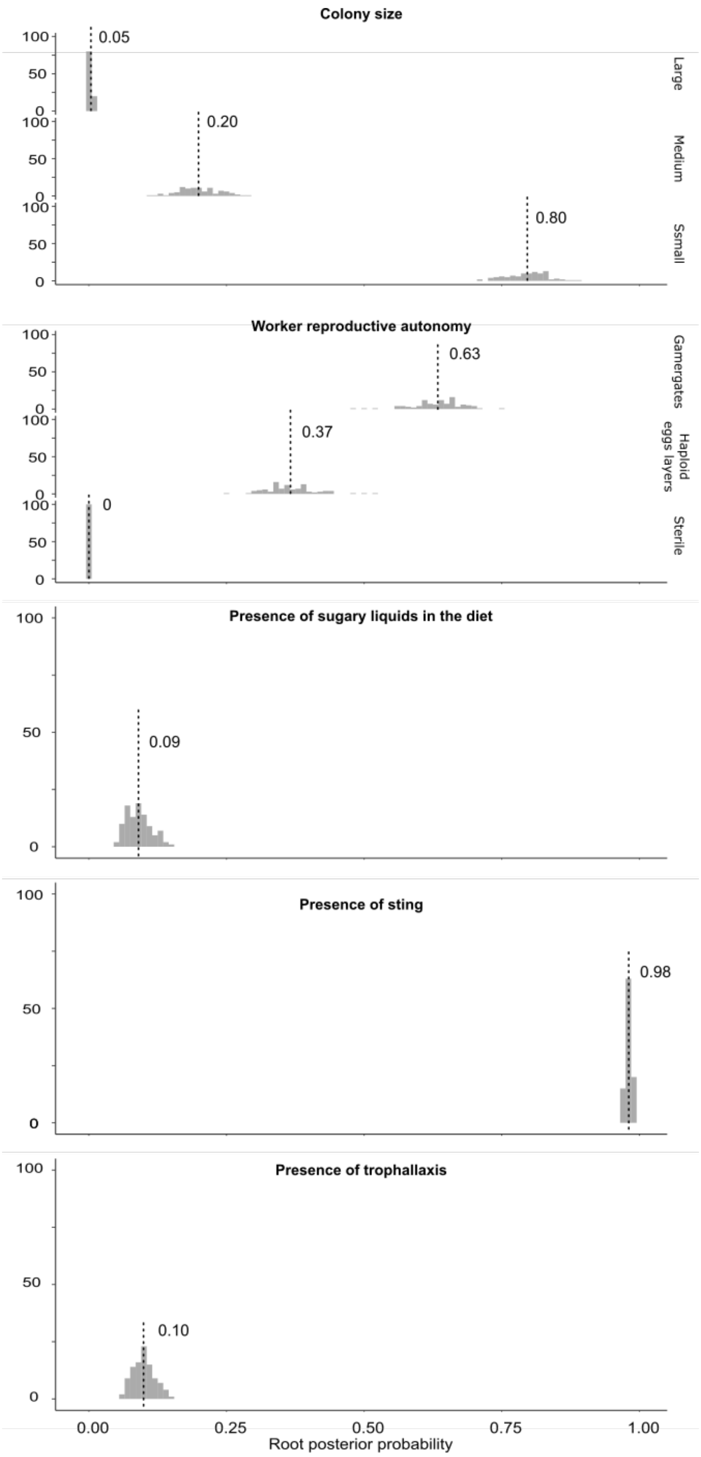

Figure S4 Histogram of the posterior probability of the root node for the analyses on 100 stem posterior trees, for each trait. Numbers indicates the mean of the posterior probabilities for each state. For traits with two states, we report only the probabilities of the presence of the trait.

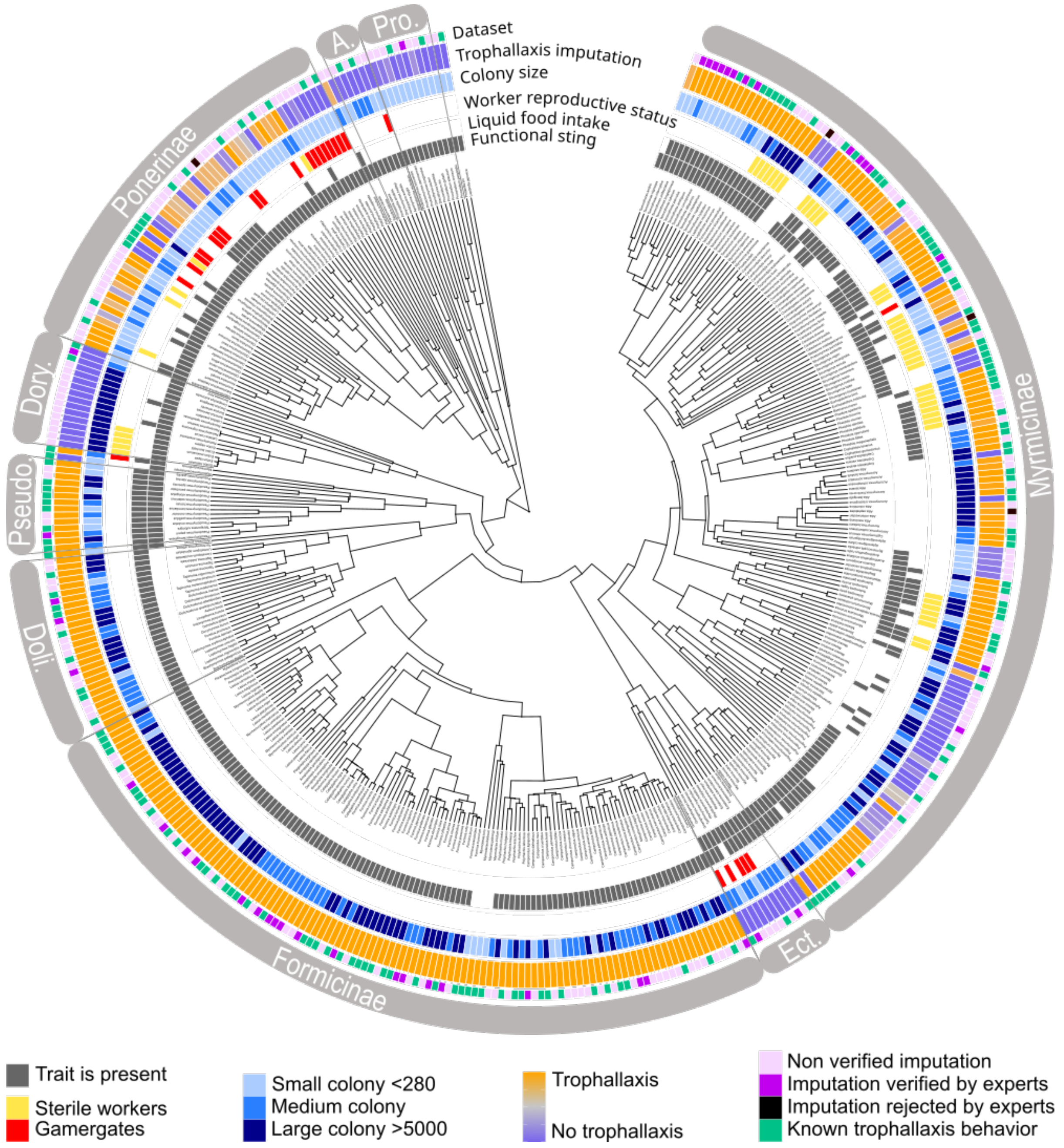

Figure S5 Overview of the traits collected per species. The inner ring identifies the presence of a functional sting in the species, the second whether ants opportunistically or extensively drink sugary liquids. The third ring displays information on workers reproductive potential, the fourth ring is associated to the size category of a colony. Finally, the fifth ring provides the imputation for stomodeal trophallaxis from the BNN, the sixth whether species has a known, imputed, verified, or corrected trophallaxis behavior and the outer ring labels most abundant subfamilies in the full dataset. Absence of functional sting, liquid food intake and non-gamergate/non-sterile workers are left white. Doli. stands for Dolichoderinae, Pseudo. for Pseudomyrmeciinae, Dory. for Dorylinae, A. for Amblyoponinae and Pro. for Proceratiinae

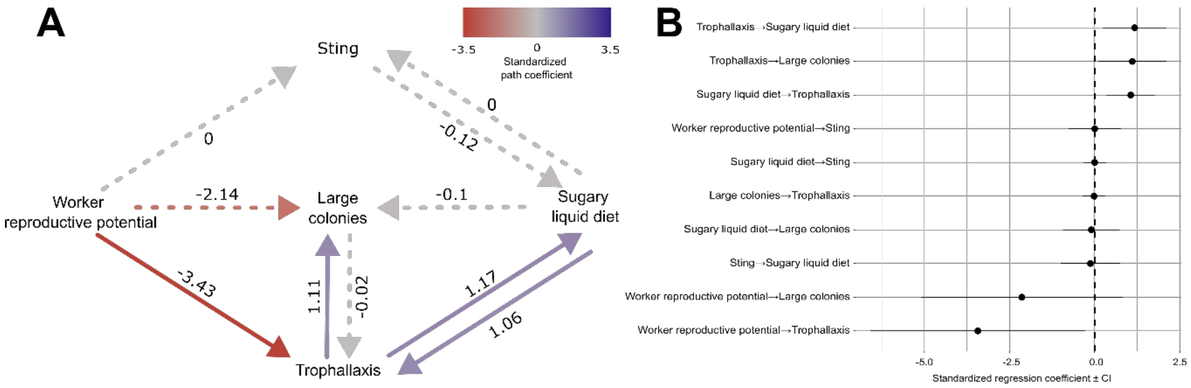

Figure S6 A) Averaged model of all four models set as an input as defined by the Phylogenetic Path Analysis. Dashed lines indicate non significance, and colors correspond to the value of the standardized path coefficient. B) Representation of 95% confidence intervals for each relationship between traits.

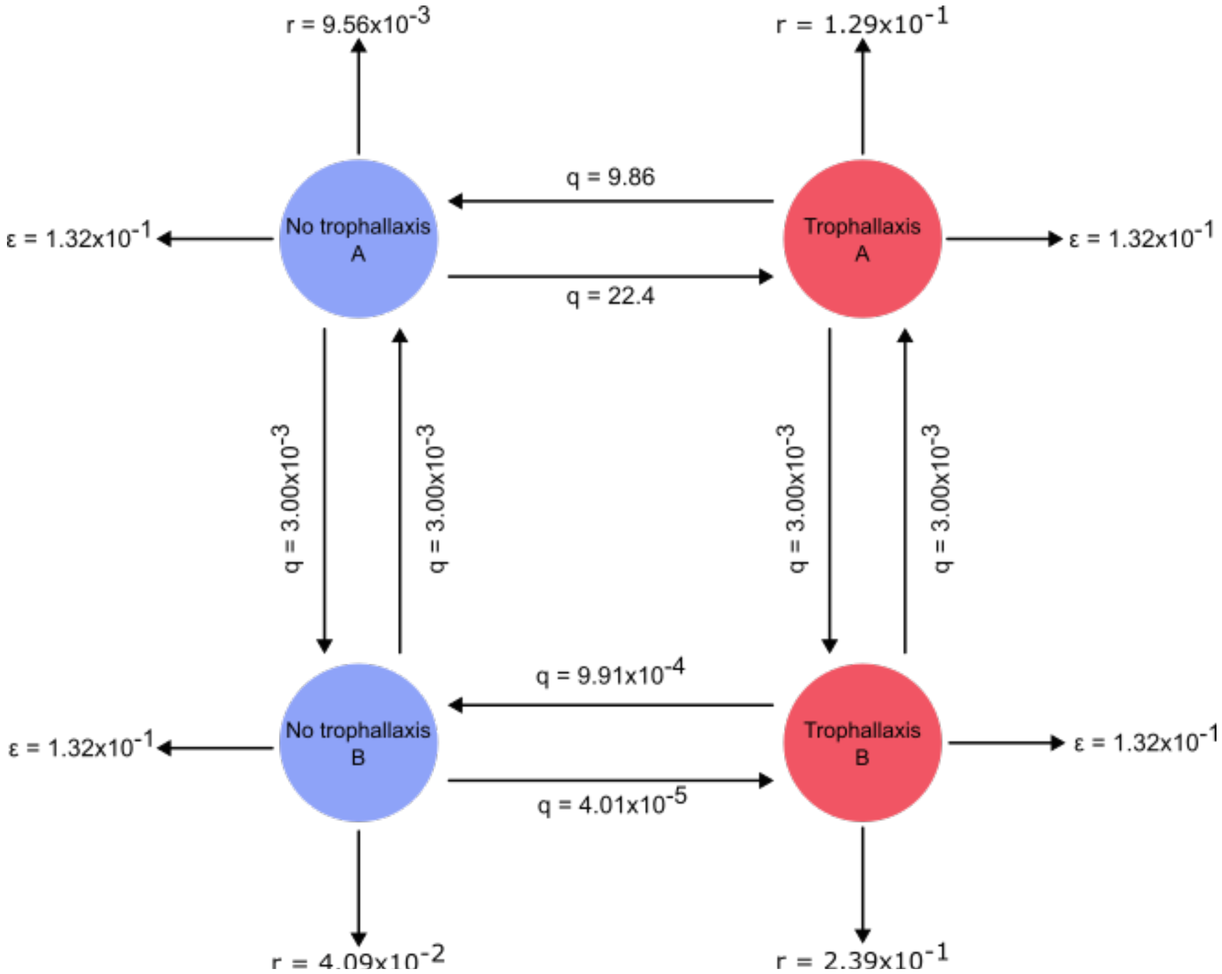

Figure S7 Representation of the character independent HiSSE model with two hidden states. q indicates transition rates between state combinations, μ is extinction, λ is speciation, net diversification rate r is calculated as $r = \lambda - \mu$, T is the Turnover, calculated as $T = \lambda + \mu$, ε is the extinction fraction, calculated as $\varepsilon = \mu / \lambda$.

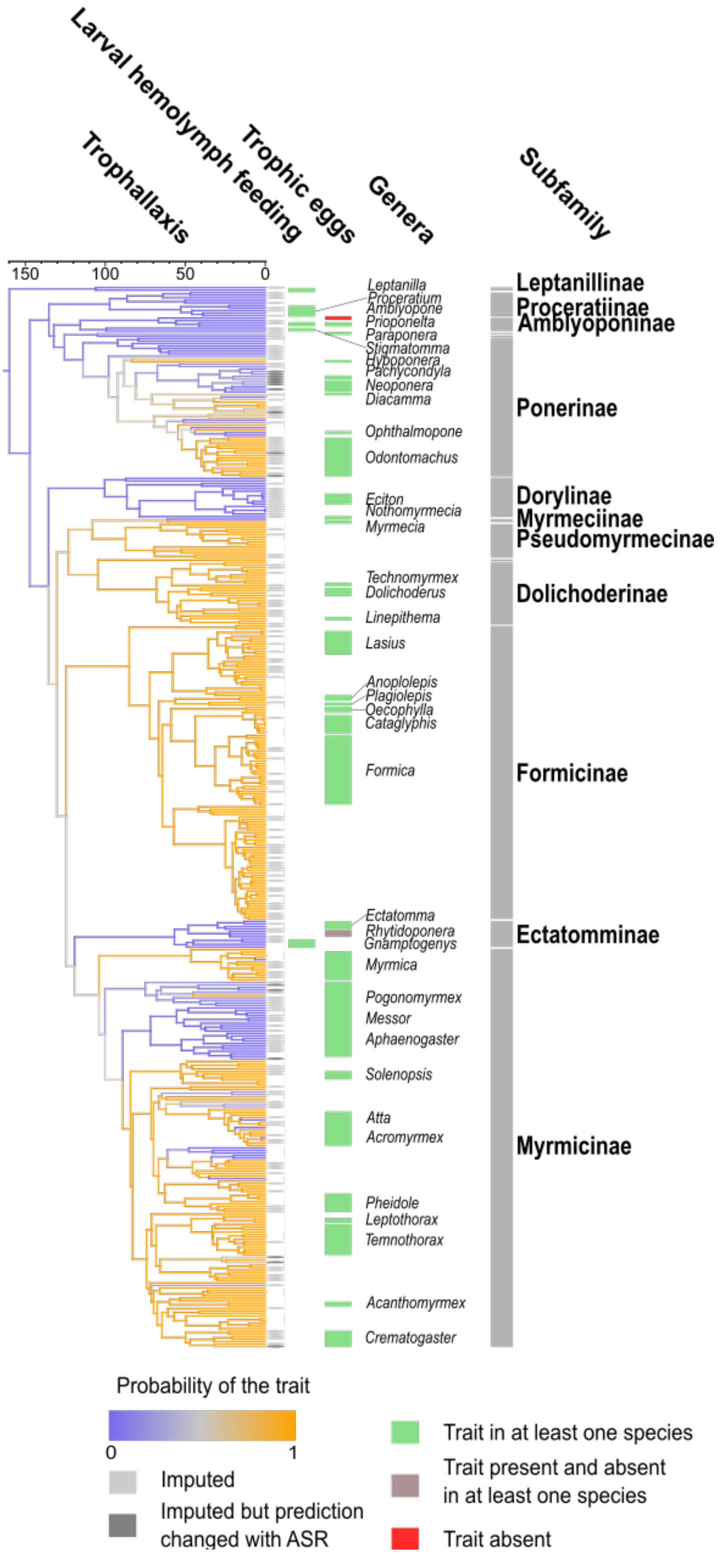

Figure S8 Records of genera in which adults drink larval hemolymph and/or produce trophic eggs. The tree represents the ancestral state reconstruction of trophallaxis on the MCC tree for 417 ant species. The branch color reflects the posterior probability of trophallaxis. We highlight whether the trophallaxis behavior of extant species are imputed (light grey). We extrapolated from species records to the genus level whether adults drink larval hemolymph and/or produce trophic eggs. In green, at least one species has the trait, in maroon, at least one species does and at least one species does not, and in red at least one species does not have the trait.

		Trophallaxis							
Dataset		Known and expert-verified trophallaxis behavior (212 sp.)				Known, expert-verified and imputed trophallaxis behavior (417 sp.)			
Status of the trait		Present		Absent		Present		Absent	
Statistic		D	PP	D	PP	D	PP	D	PP
Colony size	Small	**-4.555**	**0.033**	**4.555**	**0.033**	**-4.728**	**0.034**	**4.728**	**0.034**
	Medium	0.092	0.88	-0.092	0.88	1.701	0.22	-1.701	0.22
	Large	**4.401**	**0.033**	**-4.401**	**0.033**	2.979	0.097	-2.979	0.097
Functional sting	Present	**-5.884**	**0.039**	**5.884**	**0.039**	**-7.196**	**0.016**	**7.196**	**0.016**
	Absent	**5.884**	**0.039**	**-5.884**	**0.039**	**7.196**	**0.016**	**-7.196**	**0.016**
Sugary liquids in diet	Present	**10.978**	**0.002**	**-10.978**	**0.002**	**13.409**	**0.0005**	**-13.409**	**0.0005**
	Absent	**-10.978**	**0.002**	**10.978**	**0.002**	**13.409**	**0.0005**	**-13.409**	**0.0005**
Worker reproductive potential	Gamergates	**-3.834**	**0.046**	**3.834**	**0.046**	**-5.078**	**0.025**	**5.078**	**0.025**
	Non-Gamergate/Non-sterile	2.252	0.15	-2.252	0.15	2.416	0.13	-2.416	0.13
	Sterile	1.708	0.15	-1.708	0.15	2.74	0.079	-2.74	0.079

Table S1 D-statistic (D) and Posterior probability (PP) given by the D-test, between all traits and trophallaxis, for data with known and expert-verified trophallaxis behavior (212 sp.) and all data (417 sp.). In bold, posterior probabilities smaller than 0.05, considered to be significant.

	Average over 10 cross validations		Final model on training set		On expert-verified species	
**Accuracy**	0.89		0.94		0.91	
**Precision**	0.94		0.94		0.92	
**Recall**	0.92		0.98		0.97	
**F1-score**	0.92		0.96		0.95	
**Confusion matrices**						
**Predicted / Known**	**No trophallaxis**	**Trophallaxis**	**No trophallaxis**	**Trophallaxis**	**No trophallaxis**	**Trophallaxis**
**No trophallaxis**	2.5	0.8	26	8	5	3
**Trophallaxis**	1	11.7	2	127	1	38

Table S2: Statistics and Confusion matrices of the averaged 10 cross validations on test sets of the Bayesian Neural Network, on the training set of the final model, and on the species whose trophallaxis behavior was validated by experts.

Trait	Model name	Model representation	Akaike weight	Rates confidence interval
**COLONY SIZE**	Equal rates	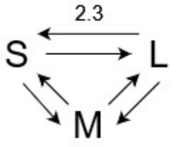	0	$R_{S \to M}$ = 2.31 95% CI [2.29—2.33]<br>$R_{S \to L}$ = 2.31 95% CI [2.29—2.33]<br>$R_{M \to S}$ = 2.31 95% CI [2.29—2.33]<br>$R_{M \to L}$ = 2.31 95% CI [2.29—2.33]<br>$R_{L \to S}$ = 2.31 95% CI [2.29—2.33]<br>$R_{L \to M}$ = 2.31 95% CI [2.29—2.33]
	All rates are different	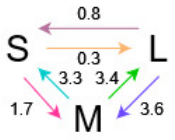	0	$R_{S \to M}$ = 1.75 95% CI [1.72—1.78]<br>$R_{S \to L}$ = 0.27 95% CI [0.26 — 0.28]<br>$R_{M \to S}$ = 3.31 95% CI [3.26 — 3.36]<br>$R_{M \to L}$ = 3.45 95% CI [3.39 — 3.51]<br>$R_{L \to S}$ = 0.83 95% CI [0.80 — 0.85]<br>$R_{L \to M}$ = 3.62 95% CI [3.57 — 3.67]
	Symmetrical	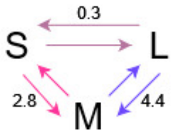	0.01	$R_{S \to M}$ = 2.84 95% CI [2.80—2.87]<br>$R_{S \to L}$ = 0.32 95% CI [0.31 — 0.34]<br>$R_{M \to S}$ = 2.84 95% CI [2.80 — 2.87]<br>$R_{M \to L}$ = 4.44 95% CI [4.39 — 4.49]<br>$R_{L \to S}$ = 0.32 95% CI [0.31 — 0.34]<br>$R_{L \to M}$ = 4.44 95% CI [4.39 — 4.49]
	Irreversible growth	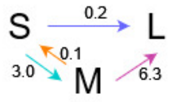	0	$R_{S \to M}$ = 2.97 95% CI [2.95—2.99]<br>$R_{S \to L}$ = 0.22 95% CI [0.22 — 0.23]<br>$R_{M \to S}$ = 0.06 95% CI [0.06 — 0.06]<br>$R_{M \to L}$ = 6.35 95% CI [6.30 — 6.40]<br>$R_{L \to S}$ = 0.05 95% CI [0.04 — 0.05]<br>$R_{L \to M}$ = 0.02 95% CI [0.02 — 0.02]

	Ordered - equal rates	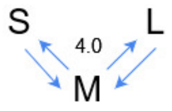	0.90	$R_{S \rightarrow M}$ = 1.80 95% CI [1.77— 1.83]<br>$R_{S \rightarrow L}$ = 0.01 95% CI [0.01 — 0.01]<br>$R_{M \rightarrow S}$ = 4.04 95% CI [3.99 — 4.08]<br>$R_{M \rightarrow L}$ = 3.61 95% CI [3.56 — 3.66]<br>$R_{L \rightarrow S}$ = 0.01 95% CI [0.01 — 0.01]<br>$R_{L \rightarrow M}$ = 4.18 95% CI [4.13 — 4.22]
	Ordered - all rates are different	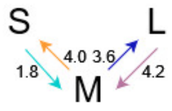	0	$R_{S \rightarrow M}$ = 1.80 95% CI [1.77— 1.83]<br>$R_{S \rightarrow L}$ = 0.01 95% CI [0.01 — 0.01]<br>$R_{M \rightarrow S}$ = 4.04 95% CI [3.99 — 4.08]<br>$R_{M \rightarrow L}$ = 3.61 95% CI [3.56 — 3.66]<br>$R_{L \rightarrow S}$ = 0.01 95% CI [0.01 — 0.01]<br>$R_{L \rightarrow M}$ = 4.18 95% CI [4.13 — 4.22]
	Ordered - symmetrical	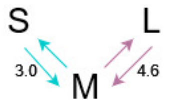	0.09	$R_{S \rightarrow M}$ = 3.03 95% CI [3.00— 3.07]<br>$R_{S \rightarrow L}$ = 0.01 95% CI [0.01 — 0.01]<br>$R_{M \rightarrow S}$ = 3.03 95% CI [3.00 — 3.07]<br>$R_{M \rightarrow L}$ = 4.58 95% CI [4.54 — 4.63]<br>$R_{L \rightarrow S}$ = 0.01 95% CI [0.01 — 0.01]<br>$R_{L \rightarrow M}$ = 4.58 95% CI [4.54 — 4.63]
	Bourke - all rates different	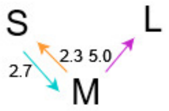	0	$R_{S \rightarrow M}$ = 2.70 95% CI [2.67— 2.74]<br>$R_{S \rightarrow L}$ = 0.01 95% CI [0.01 — 0.01]<br>$R_{M \rightarrow S}$ = 2.26 95% CI [2.23 — 2.30]<br>$R_{M \rightarrow L}$ = 4.98 95% CI [4.94 — 5.03]<br>$R_{L \rightarrow S}$ = 0.02 95% CI [0.02 — 0.02]<br>$R_{L \rightarrow M}$ = 0.02 95% CI [0.02 — 0.02]
	**Averaged Bayes model**	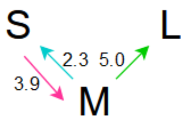	/	**$R_{S \rightarrow M}$ = 3.94 95% CI [3.91— 3.96]**<br>**$R_{S \rightarrow L}$ = 0.01 95% CI [0.01 — 0.01]**<br>**$R_{M \rightarrow S}$ = 3.94 95% CI [3.91 — 3.96]**<br>**$R_{M \rightarrow L}$ = 4.09 95% CI [4.07 — 4.12]**

				**$R_{L \to S}$ = 0.01 95% CI [0.01 — 0.02]**<br>**$R_{L \to M}$ = 4.09 95% CI [4.07 — 4.12]**
**STING**	All rates different	0 ⇄ 1 (0.4 / 0.4)	0.22	$R_{0 \to 1}$ = 0.39 95% CI [0.38 — 0.40]<br>$R_{1 \to 0}$ = 0.37 95% CI [0.36— 0.38]
	Equal rates	0 ⇄ 1 (0.3)	0.78	$R_{0 \to 1}$ = 0.35 95% CI [0.34— 0.35]<br>$R_{1 \to 0}$ = 0.35 95% CI [0.34— 0.35]
	**Averaged Baye model**	0 ⇄ 1 (0.3 / 0.4)	/	**$R_{0 \to 1}$ = 0.34 95% CI [0.33— 0.34]**<br>**$R_{1 \to 0}$ = 0.35 95% CI [0.35— 0.36]**
**TROPHALLAXIS**	All rates different	0 ⇄ 1 (0.8 / 0.4)	0.57	$R_{0 \to 1}$ = 0.43 95% CI [0.41— 0.44]<br>$R_{1 \to 0}$ = 0.76 95% CI [0.75— 0.77]
	Equal rates	0 ⇄ 1 (0.6)	0.43	$R_{0 \to 1}$ = 0.63 95% CI [0.62— 0.64]<br>$R_{1 \to 0}$ = 0.63 95% CI [0.62— 0.64]
	**Averaged Bayes model**	0 ⇄ 1 (0.6 / 0.5)	/	**$R_{0 \to 1}$ = 0.51 95% CI [0.51— 0.52]**<br>**$R_{1 \to 0}$ = 0.57 95% CI [0.57— 0.58]**
**SUGARY LIQUID FOOD IN DIET**	All rates different	0 ⇄ 1 (0.9 / 1.5)	0.51	$R_{0 \to 1}$ = 1.46 95% CI [1.44 — 1.49]<br>$R_{1 \to 0}$ = 0.88 95% CI [0.87 — 0.90]
	Equal rates	0 ⇄ 1 (1.2)	0.49	$R_{0 \to 1}$ = 1.17 95% CI [1.16 — 1.18]<br>$R_{1 \to 0}$ = 1.17 95% CI [1.16 — 1.18]
	**Averaged Bayes model**	0 ⇄ 1 (1.0 / 1.3)	/	**$R_{0 \to 1}$ = 1.32 95% CI [1.31 — 1.33]**<br>**$R_{1 \to 0}$ = 1.02 95% CI [1.0.1 — 1.03]**
**WORKERS REPRODUCTIVE AUTONOMY**	Irreversible – all rates different	F → H (3.1); H → S (0.4)	0.75	$R_{H \to S}$ = 0.39 95% CI [0.38— 0.39]<br>$R_{H \to F}$ = 0.02 95% CI [0.024— 0.02]<br>$R_{S \to H}$ = 0.02 95% CI [0.02 — 0.02]<br>$R_{S \to F}$ = 0.01 95% CI [0.01 — 0.01]<br>$R_{F \to H}$ = 3.10 95% CI [3.01—

				3.14] $R_{F \rightarrow S}$ = 0.01 95% CI [0.01— 0.01]
	Stuck – equal rates	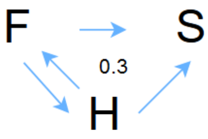	0.01	$R_{H \rightarrow S}$ = 0.34 95% CI [0.34— 0.34] $R_{H \rightarrow F}$ = 0.34 95% CI [0.34— 0.34] $R_{S \rightarrow H}$ = 0.02 95% CI [0.02 — 0.02] $R_{S \rightarrow F}$ = 0.01 95% CI [0.01 — 0.01] $R_{F \rightarrow H}$ = 0.34 95% CI [0.34— 0.34] $R_{F \rightarrow S}$ = 0.34 95% CI [0.34— 0.34]
	Stuck – all rates different	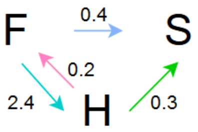	0.06	$R_{H \rightarrow S}$ = 0.33 95% CI [0.33— 0.34] $R_{H \rightarrow F}$ = 0.20 95% CI [0.19— 0.20] $R_{S \rightarrow H}$ = 0.02 95% CI [0.02 — 0.02] $R_{S \rightarrow F}$ = 0.01 95% CI [0.01 — 0.01] $R_{F \rightarrow H}$ = 2.40 95% CI [2.36— 2.45] $R_{F \rightarrow S}$ = 0.43 95% CI [0.41— 0.44]
	Reversible – equal rates	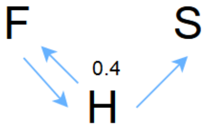	0.03	$R_{H \rightarrow S}$ = 0.35 95% CI [0.35— 0.36] $R_{H \rightarrow F}$ = 0.35 95% CI [0.35— 0.36] $R_{S \rightarrow H}$ = 0.02 95% CI [0.02 — 0.02] $R_{S \rightarrow F}$ = 0.01 95% CI [0.01 — 0.01] $R_{F \rightarrow H}$ = 0.35 95% CI [0.35 — 0.36] $R_{F \rightarrow S}$ = 0.01 95% CI [0.01 — 0.01]
	Reversible – all rates different	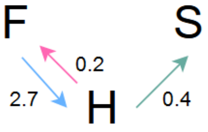	0.10	$R_{H \rightarrow S}$ = 0.37 95% CI [0.36— 0.38] $R_{H \rightarrow F}$ = 0.17 95% CI [0.16 — 0.17] $R_{S \rightarrow H}$ = 0.02 95% CI [0.02 — 0.02] $R_{S \rightarrow F}$ = 0.01 95% CI [0.01 — 0.01] $R_{F \rightarrow H}$ = 2.65 95% CI [2.60 — 2.70] $R_{F \rightarrow S}$ = 0.01 95% CI [0.01 — 0.01]

	All rates different	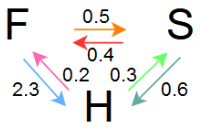	0.03	$R_{H\rightarrow S}$ = 0.35 95% CI [0.34—0.36]<br>$R_{H\rightarrow F}$ = 0.19 95% CI [0.18 —0.19]<br>$R_{S\rightarrow H}$ = 0.56 95% CI [0.54 —0.58]<br>$R_{S\rightarrow F}$ = 0.40 95% CI [0.39 —0.42]<br>$R_{F\rightarrow H}$ = 2.33 95% CI [2.28 —2.38]<br>$R_{F\rightarrow S}$ = 0.47 95% CI [0.45 —0.49]
	Equal rates	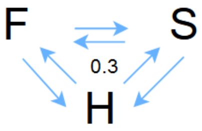	0.03	$R_{H\rightarrow S}$ = 0.32 95% CI [0.32—0.33]<br>$R_{H\rightarrow F}$ = 0.32 95% CI [0.32 —0.33]<br>$R_{S\rightarrow H}$ = 0.32 95% CI [0.32 —0.33]<br>$R_{S\rightarrow F}$ = 0.32 95% CI [0.32 —0.33]<br>$R_{F\rightarrow H}$ = 0.32 95% CI [0.32 —0.33]<br>$R_{F\rightarrow S}$ = 0.32 95% CI [0.32 —0.33]
	**Averaged Bayes model**	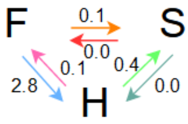	/	**$R_{H\rightarrow S}$ = 0.38 95% CI [0.38—0.39]**<br>**$R_{H\rightarrow F}$ = 0.07 95% CI [0.07 —0.07]**<br>**$R_{S\rightarrow H}$ = 0.04 95% CI [0.04 —0.04]**<br>**$R_{S\rightarrow F}$ = 0.03 95% CI [0.03 —0.03]**<br>**$R_{F\rightarrow H}$ = 2.83 95% CI [2.80 —2.86]**<br>**$R_{F\rightarrow S}$ = 0.06 95% CI [0.06 —0.06]**

Table S3 Representation of the models for each trait, (S = small colony size, M = medium colony size and L = large colony size, F = Full worker reproductive autonomy, H = haploid layers, S = sterile workers, 0 = absence of the trait, and 1 = presence of the trait) used for sMap Bayesian stochastic mapping. Arrows of the same color indicate the same rate of evolution. Numbers are the rates as estimated from sMap with a Gamma distribution as a prior on the full 417 species dataset. Akaike weights for blending models were calculated using the following equation:

$$\mathbb{P}(\text{Model} \mid \text{Data}) = \frac{\mathbb{P}(\text{Model}) \cdot \mathbb{P}(\text{Data} \mid \text{Model})}{\sum_{i=1}^{n} \mathbb{P}(\text{Model}_i) \cdot \mathbb{P}(\text{Data} \mid \text{Model}_i)}$$

where $\mathbb{P}$(Model) is the prior probability for the model and $\mathbb{P}$(Data | Model) is the marginal likelihood for that model.

Model	Turnover	Extinction fraction	AIC
BiSSE	1,2	1,1	89936.21
**HiSSE**	1,2,3,4	1,1,1,1,1	**88776.98**
HiSSE CID 2	1,1,2,2	1,1,1,1,1	89149.51

Table S4 Characteristics of the speciation and extinction models for the HiSSE analysis.

Node	Model	Fixed root state	Marginal likelihood	Bayes factor		
				Trophallaxis present – Equal rates – non-doryline formicoids node	Workers lay haploid workers – equal rates – root node	Trophallaxis present – Equal rates – Late Ponerini node
**Non-doryline Formicoids**	All rates different	Trophallaxis present	-79.42	0.71	/	/
	**Equal rates**	**Trophallaxis present**	**-79.06**	/	/	/
	All rates different	No trophallaxis	-83.66	9.19	/	/
	Equal rates	No trophallaxis	-84.34	10.57	/	/
**Root node**	All rates different	Workers that can mate	-127.46	/	1.29	/
	Equal rates	Workers that can mate	-133.79	/	13.96	/
	All rates different	Workers lay haploid eggs	-127.43	/	1.24	/
	**Equal rates**	Workers lay haploid eggs	**-126.81**	/	/	/
	All rates different	Fully sterile workers	-132.04	/	10.46	/
	Equal rates	Fully sterile workers	-133.87	/	14.11	/
**Late Ponerini node**	All rates different	Trophallaxis present	-29.9	/	/	0.18
	**Equal rates**	**Trophallaxis present**	**-29.8**	/	/	/
	All rates different	No trophallaxis	-31.3	/	/	2.86
	Equal rates	No trophallaxis	-32.2	/	/	4.70

Table S5 Summary table of the model comparisons we did on the full tree, the non-doryline formicoid and the late Ponerini subtree to measure log Bayes factor when the root node is fixed (model name), to the model with highest marginal likelihood. The bolded model and marginal likelihood indicate the model with the highest marginal likelihood.
