# Supplementary material for: Ecological change and conflict reduction led to a social circulatory system in ants
Source: Commun Biol. 2025 Feb 16;8:246. doi: 10.1038/s42003-025-07688-7 (PMC11830068; doi:10.1038/s42003-025-07688-7)
Supplement: Supplementary file 2 — Reporting summary [file 42003_2025_7688_MOESM2_ESM.pdf]

nature portfolio

Corresponding author(s): A.C. LeBoeuf

Last updated by author(s): Feb 3, 2025

Reporting Summary

Nature Portfolio wishes to improve the reproducibility of the work that we publish. This form provides structure for consistency and transparency in reporting. For further information on Nature Portfolio policies, see our Editorial Policies and the Editorial Policy Checklist.

Statistics

For all statistical analyses, confirm that the following items are present in the figure legend, table legend, main text, or Methods section.

n/a	Confirmed	
☐	☒	The exact sample size (*n*) for each experimental group/condition, given as a discrete number and unit of measurement
☐	☒	A statement on whether measurements were taken from distinct samples or whether the same sample was measured repeatedly
☐	☒	The statistical test(s) used AND whether they are one- or two-sided<br>*Only common tests should be described solely by name; describe more complex techniques in the Methods section.*
☐	☒	A description of all covariates tested
☐	☒	A description of any assumptions or corrections, such as tests of normality and adjustment for multiple comparisons
☐	☒	A full description of the statistical parameters including central tendency (e.g. means) or other basic estimates (e.g. regression coefficient) AND variation (e.g. standard deviation) or associated estimates of uncertainty (e.g. confidence intervals)
☐	☒	For null hypothesis testing, the test statistic (e.g. *F*, *t*, *r*) with confidence intervals, effect sizes, degrees of freedom and *P* value noted<br>*Give P values as exact values whenever suitable.*
☐	☒	For Bayesian analysis, information on the choice of priors and Markov chain Monte Carlo settings
☐	☒	For hierarchical and complex designs, identification of the appropriate level for tests and full reporting of outcomes
☐	☒	Estimates of effect sizes (e.g. Cohen's *d*, Pearson's *r*), indicating how they were calculated

Our web collection on statistics for biologists contains articles on many of the points above.

Software and code

Policy information about availability of computer code

Data collection	Data were collected from the literature and websites mentioned within the manuscript. Tables are provided in supplement.
Data analysis	Analyses were performed with publicly available software (links in manuscript), with sMap, and a variety of R packages all named in methods with versions. Code is made available in GitHub and Zenodo.

For manuscripts utilizing custom algorithms or software that are central to the research but not yet described in published literature, software must be made available to editors and reviewers. We strongly encourage code deposition in a community repository (e.g. GitHub). See the Nature Portfolio guidelines for submitting code & software for further information.

Data

Policy information about availability of data

All manuscripts must include a data availability statement. This statement should provide the following information, where applicable:

- Accession codes, unique identifiers, or web links for publicly available datasets
- A description of any restrictions on data availability
- For clinical datasets or third party data, please ensure that the statement adheres to our policy

We provide all our datasets and code to conduct these analyses in the Zenodo repository: https://doi.org/10.5281/zenodo.14787967 and GitHub repository https://github.com/Social-Fluids-Lab/Macroevolution_of_trophallaxis2025.

Human research participants

Policy information about studies involving human research participants and Sex and Gender in Research.

Reporting on sex and gender	N/A
Population characteristics	N/A
Recruitment	N/A
Ethics oversight	N/A

Note that full information on the approval of the study protocol must also be provided in the manuscript.

Field-specific reporting

Please select the one below that is the best fit for your research. If you are not sure, read the appropriate sections before making your selection.

☐ Life sciences ☐ Behavioural & social sciences ☒ Ecological, evolutionary & environmental sciences

For a reference copy of the document with all sections, see nature.com/documents/nr-reporting-summary-flat.pdf

Ecological, evolutionary & environmental sciences study design

All studies must disclose on these points even when the disclosure is negative.

Study description	Macroevolutionary study testing the correlational and causal interaction of behavioral, morphological and ecological traits over the ant phylogeny
Research sample	Data were collected for 416 ant species. These species were chosen according to data availability and to acquire optimal taxon sampling for our traits of interest. Percentages of species according to subfamily are reported in the Supplement.
Sampling strategy	For true trophallaxis data, we used everything found in the literature. For imputed data we chose to only impute to achieve an approximately equal number of imputed species as known.
Data collection	Authors MPM and AL searched the literature. The database on trophallaxis is available at https://osf.io/cuapw/
Timing and spatial scale	Data on trophallaxis were collected throughout 2020, data on other traits throughout 2021.
Data exclusions	Data were excluded if species were not found in the phylogenetic tree we used (Economo et al. 2018) and thus could not be included.
Reproducibility	We used multiple training and test-set splits and ran our analyses on the MCC tree and 100 posterior trees.
Randomization	We used multiple training and test-set splits and ran our analyses on the MCC tree and 100 posterior trees.
Blinding	N/A

Did the study involve field work? ☐ Yes ☒ No

Reporting for specific materials, systems and methods

We require information from authors about some types of materials, experimental systems and methods used in many studies. Here, indicate whether each material, system or method listed is relevant to your study. If you are not sure if a list item applies to your research, read the appropriate section before selecting a response.

Materials & experimental systems

n/a	Involved in the study
☒	☐ Antibodies
☒	☐ Eukaryotic cell lines
☒	☐ Palaeontology and archaeology
☒	☐ Animals and other organisms
☒	☐ Clinical data
☒	☐ Dual use research of concern

Methods

n/a	Involved in the study
☒	☐ ChIP-seq
☒	☐ Flow cytometry
☒	☐ MRI-based neuroimaging
